# Adherens junction protein expression is associated with poor response to neoadjuvant FLOT chemotherapy and pro‐inflammatory tumor microenvironment in esophageal adenocarcinoma

**DOI:** 10.1002/ijc.70426

**Published:** 2026-03-05

**Authors:** Bastian Grothey, Heike Löser, Tillmann Bedau, Wolfgang Schröder, Christiane J. Bruns, Thomas Zander, Max Krämer, Reinhard Büttner, Alexander Quaas

**Affiliations:** ^1^ Institute of Pathology, Faculty of Medicine and University Hospital of Cologne, University of Cologne Cologne Germany; ^2^ Department of General Visceral and Cancer Surgery, Faculty of Medicine and University Hospital of Cologne Cologne Germany; ^3^ Department I of Internal Medicine Center for Integrated Oncology (CIO), University of Cologne Cologne Germany

**Keywords:** cell adhesion, esophageal adenocarcinoma, FLOT perioperative treatment, tumor microenvironment

## Abstract

Esophageal adenocarcinoma (EAC) demonstrates poor survival rates despite multimodal treatments, with significant inter‐patient variability in response to standard pre‐/perioperative therapies. This study investigates the relationship between adherens junction (AJ) protein expression in pre‐treatment biopsies and response to neoadjuvant therapies in EAC. Mass spectrometry‐based proteomics was performed on diagnostic biopsies from 157 EAC patients who subsequently received either perioperative FLOT chemotherapy or neoadjuvant CROSS radiochemotherapy. Protein expression profiles were analyzed with emphasis on AJ components. Findings were correlated with treatment response and histopathological parameters. We identified a distinct protein cluster enriched for AJ components that significantly correlated with response to FLOT chemotherapy. Among FLOT major responders, 94% exhibited high AJ expression. Low AJ expression occurred across histological subtypes, with varying frequencies: 27% in tubular‐intestinal, 40% in mixed‐type, and 50% in diffuse carcinomas. Low AJ expression was significantly associated with a pro‐inflammatory tumor microenvironment and extracellular matrix (ECM) remodeling. AJ protein expression represents a potential predictive parameter for FLOT chemotherapy response in EAC and defines distinct phenotypes. The association between reduced AJ expression and inflammatory features suggests these tumors may represent candidates for further investigation of targeted therapeutic approaches, though the role of immunotherapy in this context remains to be determined. Pre‐treatment AJ assessment could guide therapy selection to avoid ineffective therapies.

## Graphical abstract

What's new?

Esophageal adenocarcinoma (EAC) is an aggressive cancer with poor survival and variable response to perioperative chemotherapy. Although histopathological features correlate with outcomes, the relationship specifically between adherens junction (AJ) protein expression and treatment response in EAC remains uncertain. Here, AJ expression was examined in EAC patients in relation to FLOT (fluorouracil, leucovorin, oxaliplatin, docetaxel) chemotherapy response. High AJ expression was found in 94 percent of major responders. In contrast, low AJ expression—observed across histological subtypes—was associated with pro‐inflammatory tumor microenvironments and extracellular matrix remodeling. The findings identify AJ protein expression as a potential predictive marker for FLOT response.

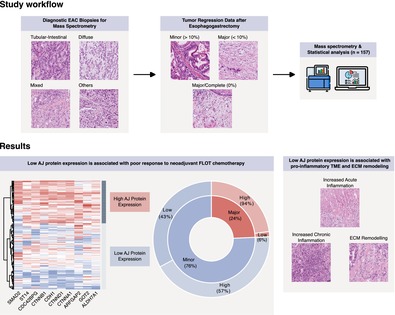

**Abbreviations**
AJ: Adherens junction
CROSS: Chemoradiotherapy for Oesophageal Cancer Followed by Surgery Study
EAC: Esophageal adenocarcinoma
ECM: Extracellular matrix
EMT: Epithelial‐mesenchymal transition
FLOT: Fluorouracil, Leucovorin, Oxaliplatin, Docetaxel
KEGG: Kyoto Encyclopedia of Genes and Genomes
MCL: Markov Cluster Algorithm
ORA: Overrepresentation analysis
TME: Tumor microenvironment

## 1 INTRODUCTION

Esophageal adenocarcinoma (EAC) represents one of the most aggressive malignancies of the gastrointestinal tract, with rising incidence and a 5‐year survival rate below 20%.^1^, ^2^ Current standard of care for locally advanced disease incorporates neoadjuvant therapy followed by surgical resection, which has demonstrated survival benefits compared to surgery alone.^3^

Two primary pre‐/perioperative treatment regimens have emerged as standard protocols: the CROSS regimen (chemoradiotherapy) and the FLOT regimen (chemotherapy).^4^, ^5^ While these approaches have improved outcomes, treatment response varies considerably among patients, with only 10%–17% achieving major pathological responses.^4^ This heterogeneity in treatment efficacy highlights the critical need for predictive biomarkers to guide therapeutic decisions and improve patient stratification. Despite extensive research into imaging markers, expression profiles, and histological features, reliable clinical tools for treatment selection remain elusive, underscoring the complexity of the molecular mechanisms underlying therapy resistance.^6^, ^7^, ^8^, ^9^, ^10^

Histopathological characteristics have shown consistent associations with treatment outcomes in EAC. Patients with diffuse histological subtype features demonstrate inferior response to therapy and poorer survival.^9^ Even minimal diffuse subtype components in otherwise typical EAC significantly reduce pathologic complete response rates.^10^ In gastric and esophageal adenocarcinomas diffuse histological subtype is associated with loss of cell adherens junctions (AJs) protein expression, particularly E‐cadherin.^11^, ^12^, ^13^ AJs are essential cell–cell adhesion complexes that maintain epithelial integrity and are frequently dysregulated during carcinogenesis.^14^, ^15^ These junctions are partly composed of cadherins (e.g., E‐cadherin [CDH1]) and catenins (α‐catenin (CTNNA1), p120‐catenin (CTNND1), and β‐catenin (CTNNB1)), which form dynamic links between transmembrane adhesion molecules and the actin cytoskeleton. Disruption of AJ complexes has been associated with epithelial‐mesenchymal transition (EMT), enabling cancer cells to acquire migratory and invasive properties.^14^ Aberrant expression of AJ proteins is more prevalent in diffuse compared to tubular‐intestinal subtype EAC and correlates with tumor grade and stage.^11^, ^16^ However, the specific relationship between adherens junction protein expression patterns and response to different chemotherapy regimens has not been characterized. Moreover, while reduced E‐cadherin expression is a hallmark of diffuse‐type carcinomas, the broader dysregulation of the adherens junction complex across EAC histological subtypes, including the classic tubular‐intestinal subtype, requires more comprehensive investigation.

In this study, we employed mass spectrometry‐based proteomics to analyze protein expression profiles in diagnostic (pre‐treatment) EAC biopsies, focusing specifically on adherens junction proteins and their potential association with response to neoadjuvant FLOT chemotherapy or CROSS radiochemotherapy. We identified a distinct protein cluster enriched for adherens junction components that significantly correlates with FLOT treatment outcomes but not with CROSS therapy response. Consistent with previous studies, we found that AJ dysregulation extends beyond classical diffuse morphology and defines distinct phenotypes with significant therapeutic implications. Furthermore, we discovered that reduced AJ expression strongly associates with a pro‐inflammatory tumor microenvironment and extracellular matrix (ECM) remodeling, including a dysregulation of adhesion‐related ECM components. Our findings provide novel insights for developing predictive biomarkers to guide therapeutic decisions in EAC management and offer new perspectives on the tumor biological heterogeneity of these tumors.

## 2 METHODS

### 2.1 Study design and patient cohort

We analyzed formalin‐fixed paraffin‐embedded pre‐treatment endoscopically obtained biopsies from 157 patients with esophageal adenocarcinoma who underwent neoadjuvant therapy followed by surgical resection at the University Hospital Cologne. These samples were part of a larger project for mass spectrometry proteomic analysis that also included esophageal squamous cell carcinomas and normal esophageal tissue. The cohort included 72 patients (45.9%) who received FLOT chemotherapy (fluorouracil, leucovorin, oxaliplatin, and docetaxel) and 85 patients (54.1%) who underwent CROSS radiochemotherapy (chemoradiotherapy followed by surgery). Clinical and pathological characteristics were documented according to standard protocols, including TNM classification, histological subtype, lymphovascular invasion, vascular invasion, and perineural invasion. Treatment response was evaluated in post‐surgical specimens and classified as major response (<10% tumor residuals) or minor response (>10% tumor residuals) based on established tumor regression grading criteria.^17^

### 2.2 Histopathological assessment

Formalin‐fixed, paraffin‐embedded, H&E‐stained tissue sections from diagnostic biopsies were retrospectively reevaluated by a senior pathologist for specific histopathological features including: (1) morphological subtype (tubular‐intestinal, diffuse, mixed, or other); (2) ulceration/necrosis (categorized as none, few, or abundant); and (3) chronic inflammatory infiltrate characterized by lymphocytes and plasma cells (categorized as none, few, or abundant).

### 2.3 Protein extraction and mass spectrometry analysis

A detailed protocol is supplied in the Data S2. The adenocarcinoma samples analyzed in this study were part of a larger cohort for mass spectrometry proteomic analysis that also included esophageal squamous cell carcinomas and normal esophageal tissue. All samples from this comprehensive cohort were preprocessed together up to and including the analysis of protein expression quantification.

### 2.4 Protein–protein interaction network analysis

To analyze protein–protein interactions, we generated a correlation matrix using Pearson's correlation coefficient (r) based on the complete mass spectrometry expression data from our EAC patient cohort. We implemented a sequential filtering approach to refine the network. Initially, we eliminated weak interactions by removing connections with absolute correlation values below 0.4. To further streamline the network, we retained only the three strongest absolute correlations for each protein node while discarding all other interactions. For the identification of distinct protein clusters within this refined network, we employed the Markov Cluster Algorithm (MCL) using the MCL implementation package in R.^18^ Pathway enrichment analysis was performed for each cluster using clusterProfiler (KEGG) R package.^19^ K‐means clustering was applied to separate patients into groups based on adherens junction protein expression.

### 2.5 Differential expression analysis

Differential expression analysis between adherens junction (AJ) expression groups was performed using the limma package in R.^20^ Proteins with *p*‐value <0.05 and an adjusted *p*‐value <0.2 were considered differentially expressed. Pathway enrichment analysis of upregulated proteins in AJ‐low tumors was conducted using both KEGG and Reactome databases with R packages clusterProfiler and ReactomePA.^19^, ^21^ ECM components were identified and analyzed using the Matrisome database, which includes structural components of the ECM and matrisome‐associated proteins.^22^ In the following, our definition of ECM therefore encompasses proteins that interact with extracellular components, regardless of their spatial restriction to the extracellular space.

### 2.6 Distance calculations

Euclidean distances between tubular‐intestinal AJ‐low samples and other subgroups (tubular‐intestinal AJ‐high, diffuse, mixed) were calculated based on both adherens junction cluster protein expression and the complete quantified proteome. Internal distances within the tubular‐intestinal AJ‐low group were also calculated to assess homogeneity of protein expression.

### 2.7 Statistical analysis and graphical visualization

The data processing, statistical analyses and graphical visualization were conducted using the R (version 4.4.2, R Foundation for Statistical Computing, Vienna, Austria). Fisher's exact test was used to evaluate associations between categorical variables, including AJ expression patterns, histopathological features, and treatment response. Wilcoxon rank‐sum test was used to compare Euclidean distances between groups. P‐values <0.05 were considered statistically significant. Inkscape was used to create graphical illustrations (https://inkscape.org/). Additional illustrations were obtained from Flaticon (https://www.flaticon.com).

## 3 RESULTS

### 3.1 Network analysis of protein expression reveals association between adherens junction proteins and FLOT chemotherapy response

**FIGURE 1:**
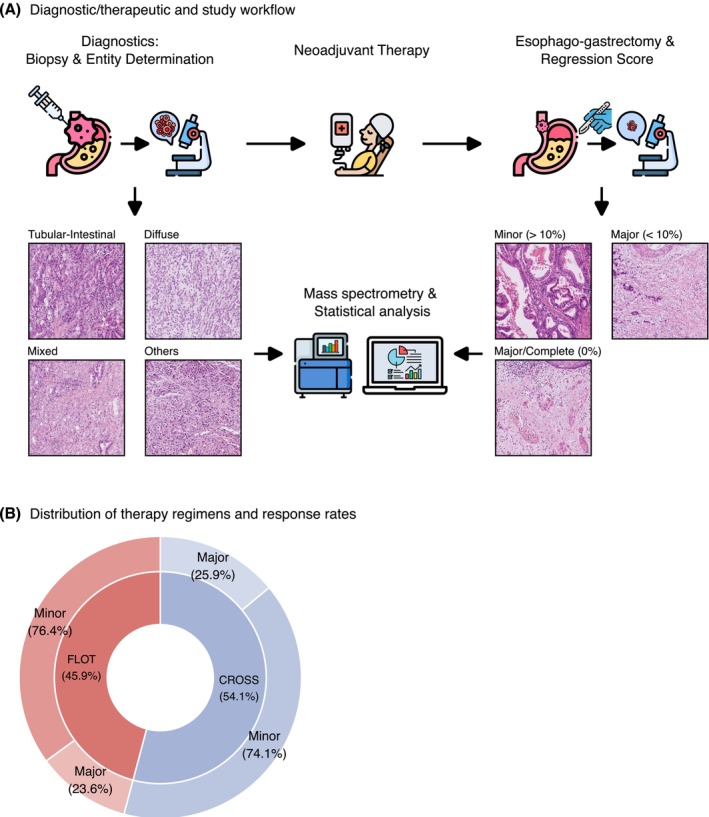
Study design and cohort composition (A) Schematic representation of the diagnostic and therapeutic workflow. Initial diagnostic biopsies obtained through endoscopy undergo pathological examination for entity determination, with cases classified as Tubular‐Intestinal, Mixed, Diffuse, or Others. These pre‐treatment biopsies were analyzed by mass spectrometry to quantify protein expression. Patients subsequently received neoadjuvant therapy, either FLOT chemotherapy or CROSS radiochemotherapy, followed by esophago‐gatstrectomy. Post‐surgical specimens were evaluated for therapy response and classified as Major (<10% tumor residuals) or Minor (>10% tumor residuals) responders based on histopathological tumor regression. This regression scoring served as the basis for correlating protein expression with therapy response, focusing on cell adhesion proteins. (B) Distribution of therapy regimens and response rates in the study cohort. The inner ring shows the distribution of neoadjuvant therapy types, while the outer ring indicates the proportion of Major and Minor responders within each therapy group. No significant differences could be between the therapy regimens and response rates (Fisher's Exact Test: *p* = 0.85; *n* = 157).

We investigated cell adhesion proteins and their potential relationship with neoadjuvant therapy outcomes in esophageal adenocarcinoma through mass spectrometry analysis of pre‐treatment biopsies. We evaluated patients who underwent neoadjuvant therapy prior to esophagogastrectomy, with tumor regression assessed according to established criteria (Figure 1A). The study population consisted of 157 individuals, with 72 patients (45.9%) receiving FLOT chemotherapy and 85 patients (54.1%) receiving CROSS radiochemotherapy (Figure 1B). We observed major responses in approximately one‐quarter of cases (24.8%, *n* = 39), with similar outcomes between treatment protocols: 23.6% (*n* = 17) for those receiving FLOT and 25.9% (*n* = 22) for those receiving CROSS. Statistical evaluation demonstrated comparable effectiveness between these therapeutic strategies (Fisher's Exact Test: *p* = 0.85). Additional clinical and pathological characteristics appear in Tables 1 and 2. As anticipated, patients with limited treatment response demonstrated more advanced TNM staging parameters in post‐treatment specimens.

**TABLE 1:** Clinicopathological characteristics of patients with major/complete versus minor FLOT therapy response.

Parameter	Total	FLOT		Fisher's exact test
		Major/Complete	Minor	
	*n* (%)	*n* (%)	*n* (%)	*p*‐value
No. of patients	72	17	55	
Gender				0.2009
Male	63 (87.5)	13 (76.5)	50 (90.9)	
Female	9 (12.5)	4 (23.5)	5 (9.1)	
Age				0.3826
Age (<65)	49 (68.1)	10 (58.8)	39 (70.9)	
Age (≥65)	23 (31.9)	7 (41.2)	16 (29.1)	
(y)pT				**0.0003**
0	4 (5.6)	4 (23.5)	0 (0)	
1	15 (20.8)	7 (41.2)	8 (14.5)	
2	11 (15.3)	2 (11.8)	9 (16.4)	
3	38 (52.8)	4 (23.5)	34 (61.8)	
4	4 (5.6)	0 (0)	4 (7.3)	
(y)pN				**0.0011**
0	29 (40.3)	13 (76.5)	16 (29.1)	
1	19 (26.4)	4 (23.5)	15 (27.3)	
2	6 (8.3)	0 (0)	6 (10.9)	
3	18 (25.0)	0 (0)	18 (32.7)	
L				8.36*10 ^**−6**^
0	40 (55.6)	17 (100)	23 (41.8)	
1	32 (44.4)	0 (0)	32 (58.2)	
V				**0.0041**
0	54 (75.0)	17 (100)	37 (67.3)	
1	18 (25.0)	0 (0)	18 (32.7)	
Pn				**0.0013**
0	45 (62.5)	17 (100)	28 (50.9)	
1	19 (26.4)	0 (0)	19 (34.5)	
Unknown	8 (11.1)	0 (0)	8 (14.5)	

*Note*: A total of 72 patients were evaluated, with 17 demonstrating major/complete FLOT response and 55 showing minor response. Fisher's exact test was used to assess statistical significance between groups. Significant differences were observed in tumor stage, nodal status, lymphovascular invasion, vascular invasion, and perineural invasion between the two response groups (*p* < 0.05).Bold print marks *p*‐value below 0.05.

Abbreviations: FLOT, Fluorouracil, Leucovorin, Oxaliplatin, Docetaxel; L, Lymphovascular invasion; Pn, Perineural invasion; pN, Histopathological nodal status; pT, Histopathological tumor stage; V, Vascular invasion.

**TABLE 2:** Clinicopathological characteristics of patients with major/complete versus minor CROSS therapy response.

Parameter	Total	CROSS		Fisher's exact test
		Major/Complete	Minor	
	*n* (%)	*n* (%)	*n* (%)	*p*‐value
No. of patients	85	22	63	
Gender				0.7336
Male	72 (84.7)	18 (81.8)	54 (85.7)	
Female	13 (15.3)	4 (18.2)	9 (14.3)	
Age				1
Age (<65)	51 (60)	13 (59.1)	38 (60.3)	
Age (> = 65)	34 (40)	9 (40.9)	25 (39.7)	
(y)pT				**1.25** * **10** ^**−8**^
0	9 (10.6)	9 (40.9)	0 (0)	
1	11 (12.9)	5 (22.7)	6 (9.5)	
2	16 (18.8)	5 (22.7)	11 (17.5)	
3	49 (57.6)	3 (13.6)	46 (73)	
4	0 (0)	0 (0)	0 (0)	
(y)pN				**0.0438**
0	39 (45.9)	14 (63.6)	25 (39.7)	
1	23 (27.1)	7 (31.8)	16 (25.4)	
2	14 (16.5)	1 (4.5)	13 (20.6)	
3	9 (10.6)	0 (0)	9 (14.3)	
L				0.0699
0	55 (64.7)	18 (81.8)	37 (58.7)	
1	30 (35.3)	4 (18.2)	26 (41.3)	
V				0.0592
0	73 (85.9)	22 (100)	51 (81)	
1	11 (12.9)	0 (0)	11 (17.5)	
Unknown	1 (1.2)	0 (0)	1 (1.6)	
Pn				**0.0002**
0	43 (50.6)	17 (77.3)	26 (41.3)	
1	23 (27.1)	0 (0)	23 (36.5)	
Unknown	19 (22.4)	5 (22.7)	14 (22.2)	

*Note*: A total of 85 patients were evaluated, with 22 demonstrating major/complete FLOT response and 63 showing minor response. Fisher's exact test was used to assess statistical significance between groups. Significant differences were observed in tumor stage, nodal status, and perineural invasion between the two response groups (*p* < 0.05). Bold print marks *p*‐value below 0.05.

Abbreviations: CROSS, Chemoradiotherapy for Oesophageal Cancer Followed by Surgery Study; L, Lymphovascular invasion; Pn, Perineural invasion; pN, Histopathological nodal status; pT, Histopathological tumor stage; V, Vascular invasion.

**FIGURE 2:**
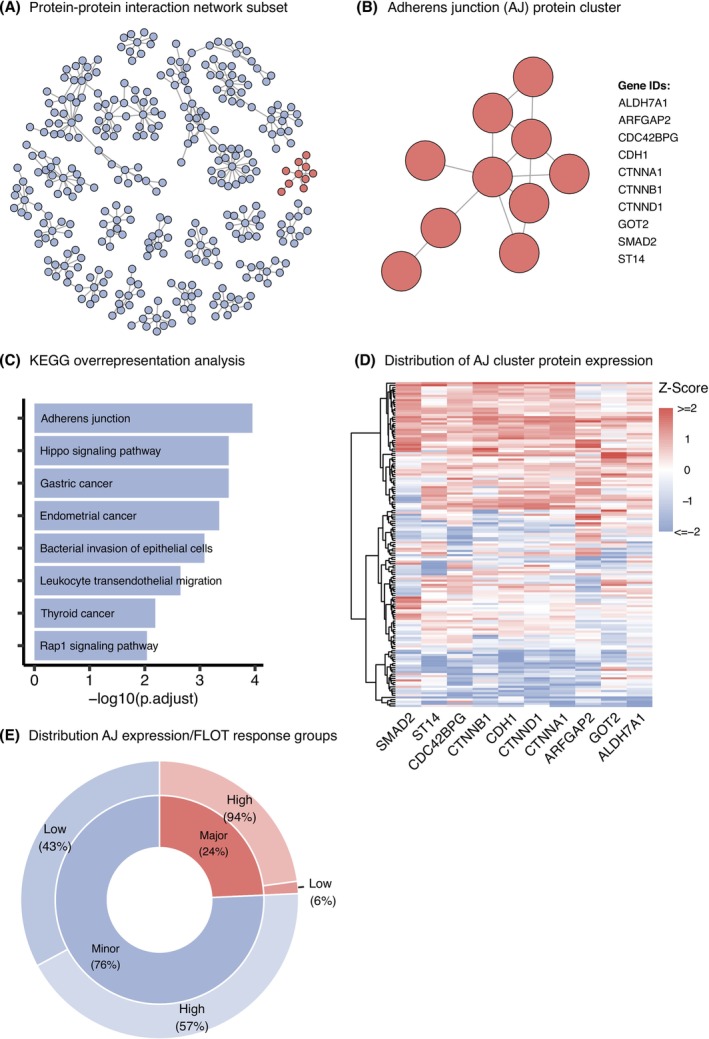
Protein–protein interaction network cluster analysis and associations to FLOT/CROSS chemotherapy response. (A) Protein–protein interaction network analysis of mass spectrometry data showing clusters with ≥10 proteins. The cluster with significant enrichment of adherens junction (AJ) proteins is highlighted in red (adjusted *p* = 0.00011). (B) Detailed view of the AJ‐enriched cluster showing the network of 10 proteins involved in the KEGG Adherens Junction pathway. Key members with established roles in cell adhesion, epithelial‐mesenchymal transition, and oncogenesis across cancer types include CDH1 (E‐cadherin), CTNNA1 (α‐catenin), CTNNB1 (β‐catenin), CTNND1 (p120‐catenin) and SMAD2. (C) Top 8 significantly overrepresented KEGG pathways in the AJ cluster, with ‐log10 of adjusted *p*‐values shown on the x‐axis. (D) Heatmap showing expression patterns of AJ cluster proteins across the patient cohort, with hierarchical clustering (ward.D2) of samples. Subsequent K‐means clustering separated the cohort into AJ‐high and AJ‐low groups. Analysis of FLOT‐treated cases revealed a significant association between AJ expression and therapy response (Fisher's Exact Test: *p* = 6.74 × 10^−3^; *n* = 70), while no significant association was found in CROSS‐treated patients (Fisher's Exact Test: *p* = 1; *n* = 83). (E) Distribution of therapy response in FLOT‐treated patients (inner ring) and corresponding AJ expression patterns (outer ring), showing underrepresentation of major responders in the AJ‐low group.

Following mass spectrometry analysis, we constructed a protein–protein interaction network based on expression data to identify functional protein groupings enriched for cell adhesion components. We concentrated our investigation on clusters containing at least 10 proteins (Figure 2A and Table S1). KEGG pathway analysis identified one cluster with notable enrichment of adherens junction (AJ) proteins (adjusted *p* = 0.00011; Table S2), which appears highlighted in Figure 2A. Detailed examination revealed this cluster contained proteins central to cell adhesion, epithelial‐mesenchymal transition, and oncogenesis (Figure 2B). These included critical components such as E‐cadherin (CDH1), α‐catenin (CTNNA1), β‐catenin (CTNNB1), p120‐catenin (CTNND1), and SMAD2, among others. The protein cluster also showed significant relationships with several additional pathways, including Hippo signaling, gastric cancer, endometrial cancer, and pathways related to bacterial epithelial invasion (Figure 2C).

Expression pattern analysis of these AJ proteins across our cohort using hierarchical clustering indicated distinct profiles forming two primary subgroups (Figure 2D). K‐means clustering separated patients into two categories characterized by either elevated (AJ‐high) or reduced (AJ‐low) expression of these junction proteins. When examining treatment outcomes separately by therapy type, we discovered a marked relationship between adherens junction protein levels and treatment effectiveness in patients who received FLOT chemotherapy (Fisher's Exact Test: *p* = 6.74 × 10^−3^; *n* = 70). In contrast, no such relationship was observed in those who underwent CROSS radiochemotherapy (Fisher's Exact Test: *p* = 1; *n* = 83). Further examination of FLOT‐treated cases revealed an informative pattern (Figure 2E). Among patients receiving FLOT therapy who showed minor response (76% of this treatment group), AJ expression patterns were relatively balanced, with 43% exhibiting low expression and 57% showing high expression. However, among major responders to FLOT, nearly all (94%) demonstrated high AJ expression, while a mere 6% showed low expression levels. These findings suggest that reduced expression of adherens junction proteins correlates with diminished responsiveness to FLOT chemotherapy in esophageal adenocarcinoma.

### 3.2 Proteomic and histopathological characterization of AJ expression groups reveals association with inflammatory features and ECM remodeling

**FIGURE 3:**
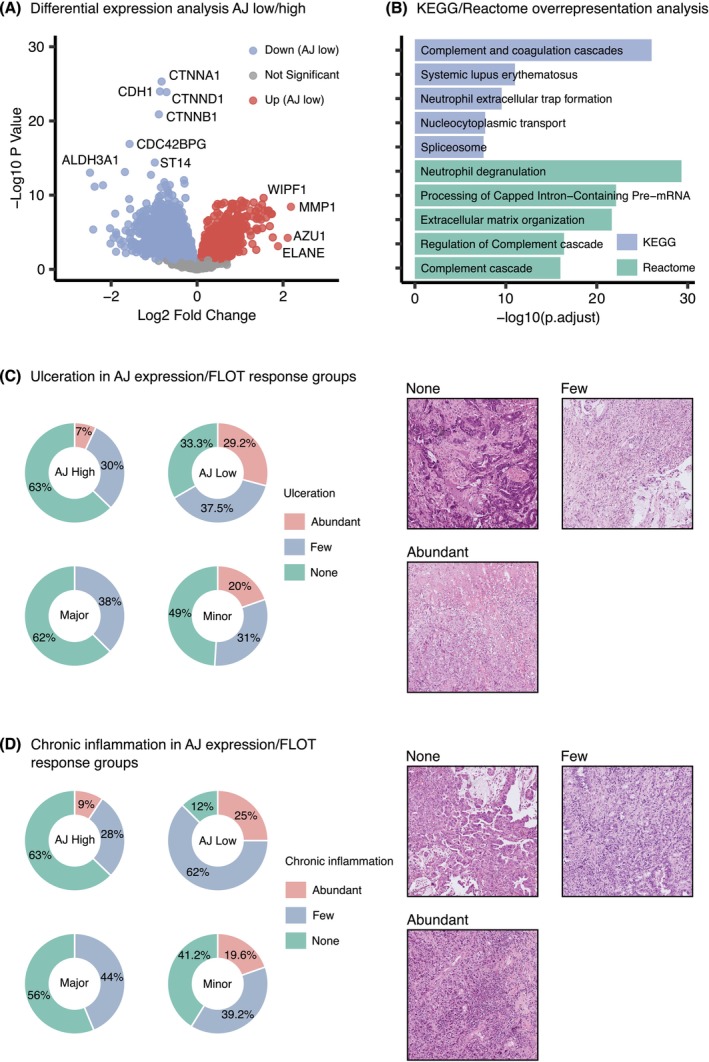
Proteomic and histopathological characterization of AJ expression groups. (A) Volcano plot showing differential protein expression between AJ‐high and AJ‐low groups (*n* = 153). Of 4492 quantified proteins, 2299 were differentially expressed (*p* < 0.05; adjusted *p* < 0.2). Negative log2 fold changes indicate downregulation in AJ‐low group (blue dots), while positive values indicate upregulation (red dots). (B) Top five overrepresented pathways from KEGG (blue) and Reactome (green) databases for proteins upregulated in AJ‐low samples (log2 fold change >0; *p* < 0.05; adjusted *p* < 0.2). Analysis revealed enrichment of innate immune system‐related pathways, including neutrophil functions and complement cascade, as well as extracellular matrix organization. (C) Distribution of ulceration severity in AJ expression groups (top) and therapy response groups (bottom) (*n* = 67). AJ‐low tumors showed significantly higher frequency of ulceration (*p* = 2.31 × 10^−2^), while abundant ulceration was exclusively observed in minor responders, though not reaching statistical significance (*p* = 1.94 × 10^−1^). Representative H&E images showing none, few, and abundant ulceration are displayed on the right. (D) Distribution of chronic inflammatory infiltrate in AJ expression groups (top) and therapy response groups (bottom) (*n* = 67). AJ‐low tumors demonstrated significantly higher levels of chronic inflammation (*p* = 1.78 × 10^−4^), while abundant chronic inflammation was exclusively observed in minor responders, though not reaching statistical significance (*p* = 1.66 × 10^−1^). Representative H&E images showing none, few, and abundant chronic inflammatory infiltrate are displayed on the right.

To better understand the proteomic distinctions between AJ expression groups, we conducted differential expression analysis comparing all quantified proteins between AJ‐high and AJ‐low tumors. From 4492 proteins identified, 2299 showed differential expression between these groups (Figure 3A and Table S3). All 10 proteins within the AJ cluster showed significant downregulation in the AJ‐low group, with key adherens junction components including CTNNA1, CDH1, CTNND1, and CTNNB1 exhibiting the most pronounced differences (each with *p*‐value <10^−20^). To characterize biological processes associated with reduced AJ expression, we analyzed proteins significantly upregulated in AJ‐low tumors through pathway analysis using both KEGG and Reactome databases (Tables S4 and S5). The most significantly enriched pathways from both analyses appear in Figure 3B. Notably, biological processes related to innate immunity were prominently represented. The most significant KEGG pathways included complement and coagulation cascades, systemic lupus erythematosus, neutrophil extracellular trap formation, nucleocytoplasmic transport, and spliceosome pathways. Complementary analysis using Reactome similarly identified neutrophil degranulation, pre‐mRNA processing, extracellular matrix organization, and complement regulation and activation as the most significantly enriched processes in AJ‐low tumors.

These proteomic findings suggested a connection between reduced AJ protein expression and enhanced inflammatory processes. To validate these observations histologically, we retrospectively evaluated H&E‐stained sections from our cohort (*n* = 67), focusing on two key features: (1) ulceration/necrosis, which typically associates with neutrophil infiltration/acute inflammation, and (2) chronic inflammatory infiltrate characterized by inflammatory round cells such as lymphocytes and plasma cells. Our histopathological analysis demonstrated a significant relationship between reduced AJ expression and increased ulceration (Fisher's exact test: *p* = 2.31 × 10^−2^, *n* = 67), as shown in Figure 3C. Most AJ‐high tumors (63%) lacked ulceration, while only one‐third of AJ‐low tumors (33.3%) were free from this feature. Furthermore, extensive ulceration was observed in 29.2% of AJ‐low tumors compared to only 7% of AJ‐high cases. When examining by treatment response, we noted that extensive ulceration appeared exclusively in minor responders (20%) and was absent in major responders, although this difference did not reach statistical significance (Fisher's exact test: *p* = 1.94 × 10^−1^, *n* = 67).

The association between AJ expression and chronic inflammation was even more pronounced (Figure 3D). Tumors with reduced AJ expression showed substantially higher levels of inflammatory infiltrate compared to those with high AJ expression (Fisher's exact test: *p* = 1.78 × 10^−4^, *n* = 67). The majority of AJ‐high tumors (63%) lacked chronic inflammatory infiltrate, while this was true for only 12% of AJ‐low tumors. Additionally, extensive chronic inflammation was present in 25% of AJ‐low tumors but in just 9% of AJ‐high cases. Similar to our observations with ulceration, extensive chronic inflammation was found exclusively in minor responders (19.6%) and was absent in major responders, though this difference was not statistically significant (Fisher's exact test: *p* = 1.66 × 10^−1^, *n* = 67).

Beyond inflammatory characteristics, we further investigated extracellular matrix organization based on our initial findings. Detailed analysis of matrix components using the Matrisome database revealed considerable differences between AJ expression groups. Among 2040 annotated extracellular matrix proteins (Table S6), 89 showed significantly higher expression in AJ‐low tumors, including Tenascin‐C (adjusted *p* = 2.74 * 10^−6^) and several collagens. In contrast, 45 matrix proteins demonstrated higher expression in AJ‐high tumors. Notably, AJ‐high tumors showed significantly higher expression of cell‐matrix adhesion regulators including the matriptase Suppressor of tumorigenicity 14 protein (ST14) (adjusted *p* = 3.05 * 10^−12^), the hemidesmosomal transmembrane collagen COL17A1 (adjusted *p* = 7.84 * 10^−5^), and Tenascin‐X (adjusted *p* = 1.30 * 10^−3^).

Collectively, these data indicate that decreased expression of adherens junction proteins associates with a pro‐inflammatory tumor microenvironment characterized by increased neutrophil activity, complement system activation, chronic inflammatory infiltration (lymphocytes and plasma cells), and extracellular matrix remodeling (e.g., upregulation of Tenascin C) with dysregulation of multiple cell adhesion systems.

### 3.3 Adherens junction protein expression defines distinct phenotypes across different histomorphological subtypes

**FIGURE 4:**
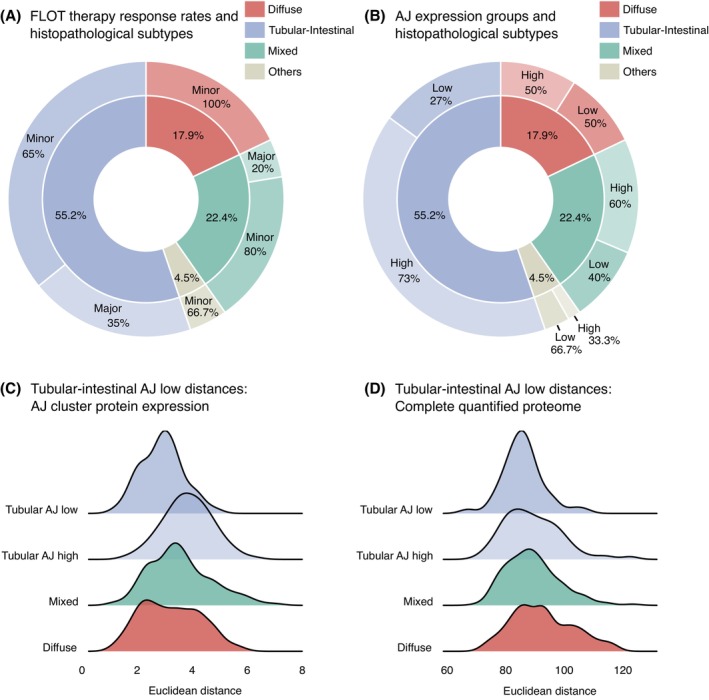
Association analysis of adherens junction expression patterns, histopathological subtypes, and chemotherapy response. (A) Distribution of chemotherapy response (outer ring: Minor = Minor responder; Major = Major responder) stratified by histopathological subtypes (inner ring) in FLOT‐treated patients. No major responders were observed in diffuse carcinomas (100% minor response). The highest proportion of major responders was found in tubular‐intestinal subtypes (35%), with a small fraction in mixed carcinomas (20%). Fisher's exact test revealed a borderline significant association between histological subtypes and therapy response (*p* = 5.58 × 10^−2^, *n* = 67). (B) Distribution of AJ expression groups (outer ring: Low = AJ low expression; High = AJ high expression) according to histopathological subtypes (inner ring). Diffuse and mixed types showed high frequencies of AJ‐low tumors (50% and 40%, respectively). Notably, 27% of tubular‐intestinal EACs also exhibited the AJ‐low phenotype. Fisher's exact test showed no significant association between histological subtypes and AJ expression groups (*p* = 2.76 × 10^−1^, *n* = 67). (C) Distribution of Euclidean distances between tubular‐intestinal AJ‐low samples (*n* = 10) and other subgroups (tubular‐intestinal AJ‐high: *n* = 27, diffuse: *n* = 12, mixed: *n* = 15) based solely on AJ cluster protein expression. In line with our previous results, tubular‐intestinal AJ‐low tumors showed significantly lower distance to diffuse cases compared to tubular‐intestinal AJ‐high tumors (Wilcoxon test, *p* = 1.25 × 10^−7^; median distances: 3.19 vs. 3.79). Significant differences were also observed between tubular‐intestinal AJ‐high vs. mixed (*p* = 9.36 × 10^−4^, median distances: 3.79 vs. 3.45) and mixed vs. diffuse subtype distance distributions (*p* = 2.46 × 10^−2^, median distances: 3.45 vs. 3.19). (D) Distribution of Euclidean distances between tubular‐intestinal AJ‐low samples (*n* = 10) and other subgroups (tubular‐intestinal AJ‐high: *n* = 27, diffuse: *n* = 12, mixed: *n* = 15) based on the entire tome. At this global level, tubular‐intestinal AJ‐low tumors showed significantly lower distances to tubular‐intestinal AJ‐high and mixed subtypes compared to diffuse carcinomas (Wilcoxon test, *p* = 2.36 × 10^−3^ and *p* = 6.4 × 10^−3^, respectively; median distances: 87.59, 87.57 vs. 91.78). No significant differences were observed between distances to tubular‐intestinal AJ‐high and mixed groups (*p* = 0.96). Notably, the internal distance between tubular‐intestinal AJ‐low samples was significantly lower than distances to tubular‐intestinal AJ‐high samples (median distances: 85.81 vs. 87.59, *p* = 9.83 × 10^−3^).

Given the established relationship between reduced adherens junction protein expression (E‐cadherin, Catenins) and diffuse‐type esophageal/gastric adenocarcinoma,^11^, ^12^, ^13^ we explored the connections between histological subtypes, AJ protein expression, and chemotherapy response in our patient cohort. The distribution of FLOT chemotherapy response rates across histological subtypes is presented in Figure 4A. In line with established knowledge,^9^, ^10^ we observed no major responders among patients with diffuse carcinomas (100% minor response). Tubular‐intestinal subtypes showed the most favorable treatment outcomes (35% major responders), with mixed‐type carcinomas demonstrating intermediate responsiveness (20% major responders). Statistical analysis indicated a borderline significant relationship between tumor morphology and therapy response (Fisher's exact test: *p* = 5.58 × 10^−2^, *n* = 67).

We next investigated the distribution of AJ expression patterns across histological subtypes (Figure 4B). Both diffuse and mixed‐type carcinomas frequently exhibited the AJ‐low phenotype (50% and 40% of cases, respectively). Interestingly, we observed that half of diffuse carcinomas maintained high AJ expression, suggesting heterogeneity within this morphological category that cannot be fully explained by AJ cluster proteins. Furthermore, consistent with the results of previous studies, the AJ‐low phenotype was not confined to diffuse and mixed subtypes, as over one‐quarter (27%) of tubular‐intestinal tumors also displayed reduced AJ protein expression. Statistical testing confirmed no significant association between histological classification and AJ expression patterns (Fisher's exact test: *p* = 2.76 × 10^−1^, *n* = 67), indicating that altered AJ expression occurs across morphological subtypes rather than being restricted to specific histological categories like the diffuse subtype.

To further explore these relationships, we calculated Euclidean distances between tubular‐intestinal AJ‐low samples and other subgroups (tubular‐intestinal AJ‐high, diffuse, mixed). Initial analysis focusing exclusively on AJ cluster protein expression (Figure 4C) revealed that tubular‐intestinal AJ‐low tumors shared greater similarity with diffuse cases than with tubular‐intestinal AJ‐high tumors when considering this specific protein subset (Wilcoxon test: *p* = 1.25 × 10^−7^; median distances: 3.19 vs. 3.79). We also observed significant differences when comparing the distances between tubular‐intestinal AJ‐low tumors and other subgroups, specifically between tubular‐intestinal AJ‐high vs. mixed (Wilcoxon test: *p* = 9.36 × 10^−4^, median distances: 3.79 vs. 3.45) and between mixed vs. diffuse subtypes (Wilcoxon test: *p* = 2.46 × 10^−2^, median distances: 3.45 vs. 3.19).

To determine whether these relationships persisted at a broader level, we expanded our analysis to include the entire quantified proteome (Figure 4D). As expected, this assessment revealed a different pattern. When considering all proteins, tubular‐intestinal AJ‐low tumors showed greater similarity to both tubular‐intestinal AJ‐high and mixed subtypes compared to diffuse carcinomas (Wilcoxon test: *p* = 2.36 × 10^−3^ and *p* = 6.4 × 10^−3^, respectively; median distances: 87.59, 87.57 vs. 91.78). We found no significant differences between distances to tubular‐intestinal AJ‐high and mixed groups (Wilcoxon test: *p* = 0.96; median distances: 87.59 vs. 87.57). Additionally, we compared internal homogeneity of protein expression within the tubular‐intestinal AJ‐low group to the distances between these tumors and AJ‐high samples of the same histological subtype. Notably, tubular‐intestinal AJ‐low tumors demonstrated greater similarity to each other (median distance: 85.81) than to tubular‐intestinal AJ‐high tumors (median distance: 87.59, *p* = 9.83 × 10^−3^), suggesting they form a distinct phenotype.

These findings show that reduced AJ protein expression represents a distinct phenotype and confirm the results of previous studies that this feature can occur across histological subtypes.^11^

## 4 DISCUSSION

Esophageal adenocarcinoma continues to present a formidable clinical challenge, with limited response to existing treatment modalities and poor overall survival rates.^1^, ^2^, ^4^ Our study provides novel insights into the determinants of therapy response by demonstrating a significant association between adherens junction (AJ) protein expression and response to FLOT chemotherapy, but not to CROSS radiochemotherapy, in EAC. Furthermore, AJ dysregulation transcends histopathological subtypes and associates with specific inflammatory characteristics in the tumor microenvironment and ECM remodeling. These findings could have important implications for treatment stratification and future therapeutic approaches in EAC management.

Our proteomic analysis identified a distinct protein cluster enriched in adherens junction (AJ) components that significantly correlates with FLOT therapy outcomes. Notably, 94% of major responders to FLOT exhibited high AJ expression, while only 6% had low expression levels, suggesting that intact AJs may be critical for FLOT chemotherapy efficacy. This association was specific to FLOT and absent in CROSS radiochemotherapy, highlighting its treatment‐specific predictive value rather than serving as a general predictive marker. The differential response patterns suggest distinct underlying mechanisms of action between these treatment modalities.

Several mechanisms might explain the relationship between AJ integrity and FLOT response. First, preserved AJs maintain vascular integrity, which could improve drug delivery by preventing vascular collapse and maintaining functional tumor perfusion.^23^, ^24^ Tumors with disrupted AJs often exhibit chaotic vasculature with poor perfusion due to elevated interstitial fluid pressure, creating a barrier to effective drug penetration.^23^, ^25^ Second, loss of AJs can lead to epithelial‐mesenchymal transition, a process associated with increased chemoresistance through multiple mechanisms including enhanced drug efflux, altered metabolism, and activation of survival signaling pathways.^26^ Third, tumors with preserved AJs may exhibit enhanced bystander effects, where chemotherapy‐induced cell death propagates to adjacent cells through maintained intercellular connections, while disrupted AJs prevent the transmission of death signals.^27^, ^28^, ^29^

The absence of association between AJ expression and CROSS response likely reflects fundamental mechanistic differences between chemoradiotherapy and systemic chemotherapy. CROSS combines direct ionizing radiation‐induced DNA damage with concurrent chemotherapy, making therapeutic efficacy less dependent on systemic drug delivery parameters such as adequate tumor perfusion, functional vasculature, and sufficient intratumoral drug retention– processes that may be compromised by AJ disruption.^23^, ^24^, ^25^, ^30^, ^31^ Furthermore, while AJ loss potentially promotes chemoresistance through EMT‐associated mechanisms including upregulation of drug efflux pumps, altered drug metabolism, and activation of survival signaling pathways, radiation resistance operates through distinct mechanisms.^26^ These include enhanced DNA repair capacity, altered cell cycle checkpoint control, and upregulated antioxidant defense systems, which function largely independently of cell adhesion status.^32^, ^33^ This mechanistic distinction provides a plausible explanation for the lack of differential CROSS response between AJ‐high and AJ‐low tumors.

However, following results from the ESOPEC study, FLOT is expected to become the standard therapy for EAC, with CROSS playing a minor role, which justifies our primary focus on the association between AJ proteins and FLOT response. Our findings suggest that assessing AJ protein expression in pre‐treatment biopsies could identify patients who would likely not benefit from FLOT chemotherapy, thereby avoiding unnecessary toxicity and guiding alternative treatment strategies.

A key finding from our study is the strong association between reduced AJ expression and increased inflammatory features in the tumor microenvironment. Tumors with low AJ expression demonstrated significantly higher levels of ulceration, neutrophil‐related processes, complement activation, and chronic inflammatory cell infiltrates.

Impaired epithelial integrity due to disrupted AJs may facilitate increased exposure to tumor/luminal antigens and microbiota in the esophagus, triggering inflammatory responses characterized by neutrophil recruitment and complement cascade activation. This mechanism has been well‐described in inflammatory bowel diseases.^34^, ^35^ Our findings suggest that similar processes may be operational in EAC, potentially contributing to tumor progression and therapy resistance through cytokine and growth factor production by infiltrating immune cells that create an immunosuppressive and pro‐tumorigenic environment.^36^

The remodeling of extracellular matrix in AJ‐low tumors further supports this inflammation‐driven model, as chronic inflammation often leads to tissue restructuring and fibrosis.^37^ Importantly, our analysis revealed not only widespread upregulation of matrix proteins, but also complex dysregulation of specific adhesion‐related ECM components in AJ‐low tumors. Notable examples include: (1) decreased expression of matriptase suppressor of tumorigenicity 14 protein (ST14), which colocalizes with E‐cadherin and plays an important role in maintaining epithelial barrier integrity^38^, ^39^; (2) diminished levels of hemi‐desmosomal transmembrane collagen COL17A1, which anchors epithelial cells to the basement membrane^40^; and (3) an inverse regulation pattern of Tenascin family members, with decreased Tenascin‐X (which stabilizes tissue architecture) and increased Tenascin‐C (which promotes cell migration and has anti‐adhesive properties).^41^ Thus, rather than finding isolated defects, we observed comprehensive dysregulation of multiple adhesion systems in AJ‐low tumors that extends beyond the previously established dysregulation of cadherins and catenins.

As indicated above, the matrix remodeling and inflammatory changes observed in AJ‐low tumors may create physical and biochemical barriers to drug delivery, potentially explaining the reduced response to FLOT chemotherapy in these cases.^42^ Specifically, increased Tenascin‐C and collagen deposition might create dense fibrotic barriers that physically impede tumor perfusion.^43^, ^44^ Destabilization of the epithelial‐stromal interface through reduced COL17A1 expression and downregulation of ST14 could further facilitate EMT progression and create an environment permissive for tumor cell survival under chemotherapeutic stress.^26^, ^38^, ^39^, ^40^, ^45^, ^46^

The inflammatory phenotype associated with reduced AJ expression could have important therapeutic implications beyond conventional chemotherapy. Tumors with enhanced immune infiltration may respond differently to immunotherapeutic approaches, including immune checkpoint inhibitors, which have shown promising results in subsets of gastrointestinal cancers.^47^ While our study did not directly evaluate immunotherapy response, the identification of this inflammatory subgroup provides a rationale for investigating targeted immunotherapeutic strategies in AJ‐low EAC patients, who demonstrate a limited response to conventional FLOT chemotherapy.

However, recent evidence from the MATTERHORN trial provides important context for interpreting potential immunotherapy efficacy in this setting. This phase III trial evaluated perioperative FLOT plus durvalumab versus FLOT plus placebo in resectable gastric and gastroesophageal junction adenocarcinomas. While the combination therapy significantly improved event‐free survival in the overall cohort, subgroup analysis revealed no benefit in tumors with diffuse histology—a finding particularly relevant given that 50% of diffuse‐type EAC in our cohort exhibited low AJ protein expression.

This challenges the hypothesis that AJ‐low tumors were inherently more responsive to immune checkpoint inhibition, as one would expect a benefit from durvalumab in the diffuse‐type subgroup. However, several factors complicate this interpretation. First, histologic classification and AJ expression represent distinct biological parameters that do not fully overlap—low AJ expression does not invariably indicate diffuse histology, nor does diffuse histology universally predict AJ status. Second, the MATTERHORN trial specifically evaluated PD‐L1 blockade, which represents only one immunotherapeutic approach. The inflammatory microenvironment in AJ‐low tumors may be more amenable to alternative immunotherapy strategies not assessed in this trial. Therefore, while the MATTERHORN trial's findings in diffuse‐type tumors provide valuable context, they may not fully capture the therapeutic potential of immunotherapy in AJ‐low tumors. Further preclinical and prospective studies stratifying patients by AJ expression status, rather than histologic subtype alone, are needed to assess whether AJ‐low tumors exhibit differential immunotherapy responsiveness.

Beyond immunotherapeutic considerations, the extensive ECM remodeling observed in AJ‐low tumors suggests additional therapeutic opportunities. Therapeutic approaches targeting extracellular matrix components or ECM‐cell interactions could represent an additive strategy to enhance chemotherapeutic efficacy in AJ‐low tumors. For instance, matrix metalloproteinase inhibitors or agents targeting specific ECM components could potentially improve drug penetration and efficacy.^48^, ^49^, ^50^ Similarly, anti‐inflammatory treatments might ameliorate the pro‐tumorigenic inflammatory environment in AJ‐low tumors, potentially enhancing their sensitivity to conventional therapies.^51^

Furthermore, our study confirms observations of previous studies that reduced AJ protein expression occurs across different histological subtypes of EAC and represents a distinct phenotype.^11^ We observed low AJ expression in 50% of diffuse, 40% of mixed, and 27% of tubular‐intestinal type tumors. Subsequent protein expression‐based distance analysis revealed that while tubular‐intestinal AJ‐low tumors shared similarities with diffuse carcinomas when considering only AJ cluster proteins, they retained global proteomic profiles more aligned with other tubular‐intestinal tumors. However, tubular‐intestinal AJ‐low tumors demonstrated greater similarity to each other than to tubular‐intestinal AJ‐high tumors, strengthening the evidence that AJ‐low tumors constitute a distinct phenotype. The observation that half of diffuse carcinomas maintained high AJ protein expression further challenges the simplistic concept that diffuse histology universally correlates with a general loss of AJ proteins. These findings could have important implications for the classification and clinical management of EAC. Traditional histopathological classification systems, while valuable for initial tumor characterization, may not fully capture the biological heterogeneity that determines treatment response. Our data indicate that AJ protein expression status could serve as an independent molecular classifier that provides complementary information to histological subtyping. Rather than viewing AJ‐low status simply as a feature of diffuse‐type tumors, it may be more appropriate to conceptualize it as a distinct molecular subtype that can manifest across different morphological backgrounds. This interpretation is supported by our finding that AJ status—rather than histological classification—predicts FLOT response, suggesting that molecular characterization may be more clinically relevant than morphological features for treatment stratification.

The integration of molecular markers like AJ expression status with traditional histopathology could enhance the precision of EAC classification and facilitate more personalized treatment approaches. However, the heterogeneity within morphological subtypes points to complex regulatory mechanisms that warrant further investigation. Specifically, further studies are needed to validate the clinical significance and to address whether loss of AJ expression represents a distinct phenotype arising through specific molecular alterations or reflects a continuous process during tumor dedifferentiation.

Several limitations of our study should be acknowledged. Despite our relatively large cohort, the number of cases in specific subgroups, particularly among major responders, was limited. Validation in larger, multi‐institutional cohorts would strengthen the clinical applicability of our findings. Additionally, while our proteomic approach provided comprehensive profiling, functional studies in preclinical models are needed to establish mechanistic links between AJ protein expression, inflammatory phenotypes, and treatment response. Furthermore, prospective studies evaluating AJ protein expression as a predictive biomarker would be necessary before clinical implementation.

## 5 CONCLUSIONS

Our study identifies an association between aberrant expression of specific adherens junction proteins and poor response to neoadjuvant FLOT chemotherapy in esophageal adenocarcinoma. In addition, we revealed an association between reduced adherens junction protein expression, a pro‐inflammatory tumor microenvironment, and extracellular matrix remodeling including a complex dysregulation of adhesion‐related ECM components. Consistent with previous findings,^11^ the dysregulation of AJ expression occurs across morphological subtypes and defines distinct phenotypes. The integration of adherens junction assessment into clinical decision‐making could improve treatment stratification and patient outcomes in EAC. Future studies should focus on validating these findings in larger cohorts and exploring targeted therapeutic strategies for patients with reduced adherens junction expression, particularly immunotherapeutic approaches that may address the inflammatory phenotype of these tumors.

## AUTHOR CONTRIBUTIONS

**Bastian Grothey:** Conceptualization; funding acquisition; writing – original draft; methodology; visualization; software; formal analysis; project administration; data curation; investigation; validation. **Heike Löser:** Conceptualization; writing – review and editing; funding acquisition. **Tillmann Bedau:** Writing – review and editing. **Wolfgang Schröder:** Writing – review and editing. **Christiane J. Bruns:** Writing – review and editing. **Thomas Zander:** Writing – review and editing. **Max Krämer:** Writing – review and editing. **Reinhard Büttner:** Writing – review and editing; resources. **Alexander Quaas:** Conceptualization; investigation; funding acquisition; writing – review and editing; methodology; project administration; supervision; resources.

## FUNDING INFORMATION

This project was funded by the Manfred Stolte Stiftung, Bayreuth, Germany (Bastian Grothey, Heike Löser, Alexander Quaas). Proteomic analysis was performed by the CECAD Proteomics Facility, CECAD—Cluster of Excellence, University of Cologne, using a Q‐Exactive Exploris 480 LC MS system (INST 1856/71‐1 FUGG) funded by the Deutsche Forschungsgemeinschaft. Max Krämer was supported by the Köln Fortune Program, Faculty of Medicine, University of Cologne.

## CONFLICT OF INTEREST STATEMENT

The authors declare no competing interests related to this study.

## ETHICS STATEMENT

The study was approved by the Ethics Committee of the University Hospital Cologne (reference number protocol code 20‐1393) and conducted in accordance with the Declaration of Helsinki. Informed consent was obtained from all individual participants included in the study.

## Supporting information


**DATA S1.** Supplementary Tables.

**DATA S2.** Supporting Information.

## Data Availability

The mass spectrometry proteomics data have been deposited to the ProteomeXchange Consortium via the PRIDE partner repository with the dataset identifier PXD071328.^52^, ^53^ Preprocessed mass spectrometry data and clinicopathological annotations are publicly available on Zenodo (https://doi.org/10.5281/zenodo.17437183). Source code for data analysis performed on the preprocessed data is also available at the same repository. Further data that support the findings of this study are available from the corresponding authors upon request.
