# Supplementary material for: Adherens junction protein expression is associated with poor response to neoadjuvant FLOT chemotherapy and pro‐inflammatory tumor microenvironment in esophageal adenocarcinoma
Source: Int J Cancer. 2026 Mar 5;159(2):501–15. doi: 10.1002/ijc.70426 (PMC13193367; doi:10.1002/ijc.70426)
Supplement: Supplementary file 2 — DATA S2. Supporting Information. [file IJC-159-501-s002.pdf]

Adherens junction protein expression is associated with poor response to neoadjuvant FLOT chemotherapy and pro-inflammatory tumor microenvironment in esophageal adenocarcinoma

Bastian Grothey, Heike Löser, Tillmann Bedau, Wolfgang Schröder, Christiane J. Bruns, Thomas Zander, Max Krämer, Reinhard Büttner, Alexander Quaas

Table of Contents

Supplementary Methods

Protein Extraction and Mass Spectrometry Analysis ……………………… Page 2 - 4

Supplementary Tables (available in separate file: Supplementary_data.xlsx)

Supplementary Table S1. Annotated results of protein-protein interaction network analysis using Markov Clustering (MCL) algorithm. …………………………….. Sheet 1

Supplementary Table S2. KEGG overrepresentation analyses of clusters derived from protein-protein interaction network analysis. …………………………………….. Sheet 2

Supplementary Table S3. Detailed results of differential expression analysis. Sheet 3

Supplementary Table S4. Detailed results from KEGG pathway overrepresentation analyses of proteins that showed significant upregulation in AJ-low EAC compared to AJ-high EAC. ……………………………………………...………………… Sheet 4

Supplementary Table S5. Detailed results from Reactome pathway overrepresentation analyses of proteins that showed significant upregulation in AJ-low EAC compared to AJ-high EAC. ……………………………………………………………………... Sheet 5

Supplementary Table S6. Matrisome database proteins used for extracellular matrix analysis. ……………………………………………………………………………... Sheet 6

Protein Extraction and Mass Spectrometry Analysis

1. **FFPE-Tissue Lysis and digestion**

   100 µl of cooled phosphate buffered saline containing 1 x Halt Protease Inhibitor Cocktail (Thermo) were added to a mixture of 1.4 mm and 2.8 mm ceramic beads in 2 ml lysis tubes (Bertin). Deparaffinized FFPE specimens were added to the vials and were homogenized with two 20 sec cycles at 5800 rpm in a precooled Precellys ball mill (Bertin). After homogenization the tubes were centrifuged for 5 min at 20.000 x g, 4°C. 80 µl supernatant were transferred to 96 well PCR plates (Armadillo, Thermo Fisher Scientific). After addition of 30 µl 20 % SDS the plates were heated to 95 °C for 10 min in a Thermocycler and sonificated using a Bioruptor (Diagenode) with 10 x 30 sec pulses and 30 sec delays between pulses. The heating and sonification sequence were repeated one more time. 6 ul 100 mM DTT and 6 ul 400 mM chloroacetamide in water were added and samples were incubated for 30 min at 55°C in a Thermocycler. 100 µl of protein lysates were used for protein digestion according to the single-pot solid phase enhanced sample preparation protocol (Hughes 2019). Hydrophilic and hydrophobic Sera-Mag carboxylate-modified magnetic particles (GE Healthcare) were combined 1:1, washed two times with 10 volumes of water and stored at 10 µg/µl. 2 µl of the bead mix were added to the samples and acetonitrile (ACN) was added to a final percentage of 50%. The mixture was incubated at room temperature for 8 min and placed on a magnetic rack for 2 min. Supernatants were removed and the beads were washed twice with 200 µl of 70 % Ethanol (EtOH) and once with 100 % ACN. The beads were air dried and reconstituted in 25 µl 50mM Triethylammonium bicarbonate (TEAB) containing 1 µg Trypsin and 0.5 µg LysC. Proteins were digested for 16 h at 37°C in a Thermocycler. Finally, the samples were acidified with 3 ul 10 % formic acid and placed on a magnetic rack. After 3 min the supernatants were transferred to fresh plates. 5 µl digest were diluted with 15 ul 0.1 % formic acid and loaded on Evotips (Evosep) according to the manufacturer instructions.

2. **Mass spectrometry**

   Samples were analyzed on a Q Exactive Exploris 480 (Thermo Scientific) mass spectrometer equipped with a FAIMSpro differential ion mobility device that was coupled to an Evosep One nano HPLC (Evosep). All samples were analyzed using the predefined 15SPD method with a Performance 15 cm × 150 µm C-18 column (Evosep)

and a 30 µm i.D. stainless steel emitter. The column was maintained at 40°C in a column oven (Sonation). The buffer system consisted of 0.1 % formic acid in water (buffer A) and B 0.1 % formic acid in acetonitrile (buffer B). The FAIMS pro was operated at -50V compensation voltage and electrode temperatures of 99.5 °C for the inner and 85 °C for the outer electrode. Identical HPLC settings were used for library generation and sample runs. The FAIMS pro was operated at -50V compensation voltage and electrode temperatures of 99.5 °C for the inner and 85 °C for the outer electrode. To generate a spectrum library by gas phase fractionation, aliquots from all samples were pooled and the pool was measured in six narrow window DIA runs covering the range from 400 m/z to 1000 m/z in six consecutive 100 m/z gas phase fractions (GPF) (Searle 2020). MS1 scans of the respective 100 m/z gas phase fraction were acquired at 60k resolution. Maximum injection time was set to 118 msec and the AGC target to 300%. MS2 scans were acquired in 25 x 4 m/z staggered windows with 60 k resolution, 118 msec maximum injection time and an AGC target of 1000%. All scans were stored as centroid. For the samples runs MS1 scans were acquired from 390 m/z to 1010 m/z at 60k resolution. Maximum injection time was set to 118 msec and the AGC target to 1000%. MS2 scans ranged from 250 m/z to 1500 m/z and were acquired at 60 k resolution using 60 x 10 m/z windows with 1 m/z overlap. Maximum injection time was 118 msec, AGC target 1000%. All scans were stored as centroid.

3. **Data processing**

The gas phase fractionation raw files were demultiplexed and transformed to mzML files using the msconvert module in Proteowizard. A predicted spectral library was generated using a human canonical Swissprot fasta and DIA-NN 1.8.1 (Demichev 2020) using the following settings: Trypsin, up to 1 missed cleavage, precursor ion range 400 m/z – 1000 m/z, fragment ion range 250 m/z – 1500 m/z, charge states 2+, 3+ and 4+. Default settings were used for the other parameters. The resulting library (20335 proteins, and 20239 genes and 2348517 precursors) was searched with the 6 GPF runs to generate a project specific library (10912 proteins, 10894 genes and 74024 precursors) using the same parameters as for prediction and 1 % FDR filtering. Sample files were searched using DIA-NN 1.8.1 with the project library. In addition to the settings used for library generation, grouping on protein names from fasta file, relaxed protein inference and retention time dependent normalization were used.

4. Data Analysis

Identifier and quantitative columns were extracted from the main DIA-NN output file and filtered. Normalised precursor quantities were used for MaxLFQ protein quantification in the DIANN R-package. Sample runs with precursor ID numbers above or above below +/- inter quartile range (13 out of 308) were regarded as outliers and removed. The protein LFQ intensities were imported into Perseus 1.6.15.0 and filtered for at least 30% data completeness. Missing values were imputed with random values from the lower end of the intensity distribution using Perseus defaults.

Literature

Hughes, C.S., Moggridge, S., Müller, T. *et al.* Single-pot, solid-phase-enhanced sample preparation for proteomics experiments. *Nat Protoc* **14,** 68–85 (2019). https://doi.org/10.1038/s41596-018-0082-x

Demichev, V., Messner, C.B., Vernardis, S.I. *et al.* DIA-NN: neural networks and interference correction enable deep proteome coverage in high throughput. *Nat Methods* **17,** 41–44 (2020). https://doi.org/10.1038/s41592-019-0638-x

Searle, B.C., Pino, L.K., Egertson, J.D. *et al.* Chromatogram libraries improve peptide detection and quantification by data independent acquisition mass spectrometry. *Nat Commun* **9,** 5128 (2018). https://doi.org/10.1038/s41467-018-07454-w
